# First Encounters: Effects of the Microbiota on Neonatal Brain Development

**DOI:** 10.3389/fncel.2021.682505

**Published:** 2021-06-08

**Authors:** Aviva Gars, Nicole M. Ronczkowski, Benoit Chassaing, Alexandra Castillo-Ruiz, Nancy G. Forger

**Affiliations:** ^1^Neuroscience Institute, Georgia State University, Atlanta, GA, United States; ^2^INSERM U1016, Team “Mucosal Microbiota in Chronic Inflammatory Diseases”, CNRS UMR 8104, Université de Paris, Paris, France

**Keywords:** neonatal, microbiota, human, mouse, neural, cell death, microglia, maternal

## Abstract

The microbiota plays important roles in host metabolism and immunity, and its disruption affects adult brain physiology and behavior. Although such findings have been attributed to altered neurodevelopment, few studies have actually examined microbiota effects on the developing brain. This review focuses on developmental effects of the earliest exposure to microbes. At birth, the mammalian fetus enters a world teeming with microbes which colonize all body sites in contact with the environment. Bacteria reach the gut within a few hours of birth and cause a measurable response in the intestinal epithelium. In adults, the gut microbiota signals to the brain via the vagus nerve, bacterial metabolites, hormones, and immune signaling, and work in perinatal rodents is beginning to elucidate which of these signaling pathways herald the very first encounter with gut microbes in the neonate. Neural effects of the microbiota during the first few days of life include changes in neuronal cell death, microglia, and brain cytokine levels. In addition to these effects of direct exposure of the newborn to microbes, accumulating evidence points to a role for the maternal microbiota in affecting brain development via bacterial molecules and metabolites while the offspring is still *in utero*. Hence, perturbations to microbial exposure perinatally, such as through C-section delivery or antibiotic treatment, alter microbiota colonization and may have long-term neural consequences. The perinatal period is critical for brain development and a close look at microbiota effects during this time promises to reveal the earliest, most primary effects of the microbiota on neurodevelopment.

## Introduction

The womb has long been assumed to be sterile, with the first direct exposure to microbes occurring at birth. Recently, this concept has been questioned, with reports of a microbial signature in the placenta, amniotic fluid, and fetal gut (e.g., Rackaityte et al., 2020). However, other studies do not see offspring-associated microbiota in healthy pregnancies distinct from contaminating DNA, hence supporting the “sterile womb” hypothesis (e.g., de Goffau et al., 2019; reviewed in Walter and Hornef, 2021). Regardless of this debate’s outcome, all agree that microbes from maternal and environmental sources rapidly and densely colonize the neonate at birth.

Although microbes colonize all body surfaces in contact with the environment, over 98% of our body’s microbes are located within the gastrointestinal tract. The gut microbiota has effects on the brain and behavior in adulthood, as demonstrated most directly by studying animals with absent or a reduced microbiota throughout life. Germ-free (GF) mice, for example, have alterations in social behavior, stress responding, cognition, and other functions (Cryan and Dinan, 2012). Some of these effects can be corrected with exposure to a microbiota in adolescence, but others persist (e.g., Clarke et al., 2013), suggesting that early life is a sensitive period for effects of microbe exposure on the brain.

In this review, we focus on microbe exposure in the immediate peri-partum period to address key questions and identify gaps in our knowledge related to: (1) how microbes signal to the neonatal brain, (2) which microbes first colonize the gut and exactly when that occurs, (3) what the effects are of microbe exposure on the neonatal brain, and (4) whether effects of microbe exposure on brain development begin *in utero*.

## What Pathways Signal the Arrival of the First Microbes?

**FIGURE 1:**
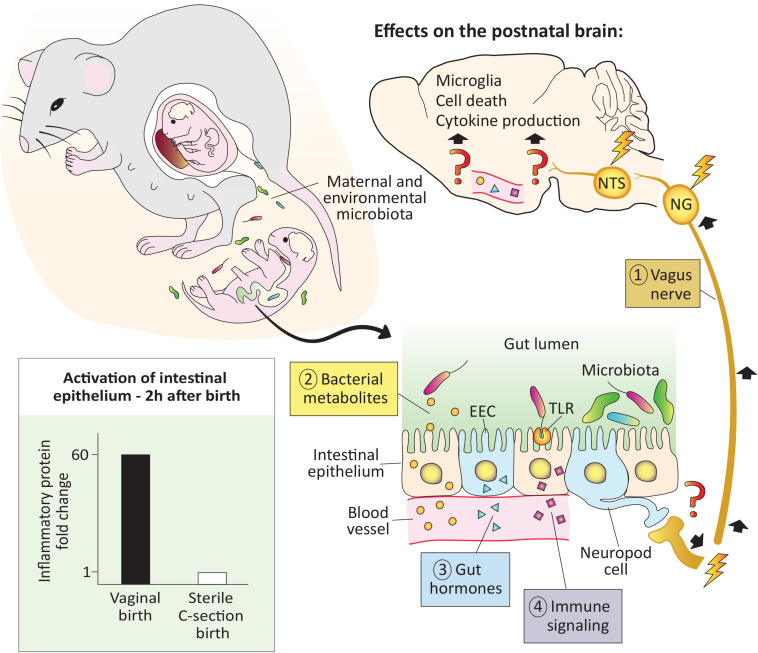
The pioneer microbiota exerts rapid effects on the neonatal brain. At birth the newborn is colonized by maternal and environmental microbiota. In mice, microbial colonization of the gut is associated with activation of the intestinal epithelium (bottom left), as early as 2 h after a vaginal but not a sterile C-section birth (see Lotz et al., 2006). In adults, microbiota signal to the brain via the vagus nerve, bacterial metabolites, gut hormones, and immune signaling, but whether these pathways are functional in the newborn remains to be demonstrated (denoted with question marks). Regardless, effects of microbes on the brain are seen within 12–14 h after birth, and include changes in cell death, microglial cell number and physiology, as well as cytokine expression (top and right; Castillo-Ruiz et al., 2018). EEC, enteroendocrine cell; NG, nodose ganglia; NTS: nucleus of the solitary tract; TLR, toll-like receptor.

Although the gut microbiota is comprised of bacteria, viruses, fungi, and protozoa, bacteria have received by far the most attention to date. In adults, gut bacteria communicate with the brain in at least four ways: via direct neural connections, bacterial metabolites, hormones, and immune signaling. Much less is known about gut-brain signaling in neonates (Figure 1), but we have at least a rudimentary understanding of what pathways are operational during this period.

### The Vagus Nerve Establishes Connections With the Gut Prenatally

The vagus nerve, which innervates the intestines from the proximal duodenum to the distal descending colon, is a major bidirectional communication system between the gut and brain. Primary sensory neurons of the vagus reside in the nodose ganglion and send a peripheral projection to the gut wall and a central projection to the nucleus of the solitary tract (NTS) in the hindbrain. The NTS, in turn, projects to several forebrain areas, such as the paraventricular nucleus of the hypothalamus (PVN) and arcuate nucleus, conveying messages related to the chemical contents of the gut, intestinal distension and inflammation, gut hormone release, and other information (Browning et al., 2017; Fülling et al., 2019). In mice, vagal sensory fibers innervate the duodenum by embryonic day (E) 14 and the distal small intestine by E16 (Ratcliffe et al., 2011). Thus, vagal connections are in place to convey the earliest information from gut microbiota to the brain. It has yet to be demonstrated, however, whether bacterial signals from the gut lumen signal to the brain via the vagus nerve in the first hours or days after birth.

Vagal innervation of the gastrointestinal tract promotes the proliferation of enteroendocrine cells (EECs), which are specialized cells in the intestinal epithelium capable of sensing the presence of microbes and relaying that information to the brain (Buchanan and Bohórquez, 2018). A subset of EECs termed neuropod cells are electrically excitable and directly synapse with vagal nerve endings. Neuropod cells can relay information from the gut to the nodose ganglion in milliseconds, which is faster than any previously known mechanism (Kaelberer et al., 2018). Although EECs are present on the day of birth in newborn rodents (Penkova et al., 2010), more work is needed to understand whether the neuropod cell-to-vagus connection is present and functional at this age.

### Microbial Metabolites Signal to the Brain

Hundreds of bacterial metabolites penetrate host body tissues (Uchimura et al., 2018), and some of these, such as aryl hydrocarbon receptor ligands, short-chain-fatty acids (SCFAs), tryptophan, and secondary bile acids have demonstrable effects on the host (e.g., Arpaia et al., 2013; Gomez de Agüero et al., 2016). SCFAs (e.g., acetate, propionate, and butyrate) have been especially well studied for their role in the gut-brain connection and are produced by bacteria as end products of the fermentation of indigestible dietary fibers. SCFAs can be shuttled across the gut epithelium by monocarboxylate transporters and act locally on the vagus nerve (Silva et al., 2020). They also can cross the blood brain barrier (BBB) where they may bind to free fatty acid receptors expressed in the brain or act via epigenetic mechanisms (Berni Canani et al., 2012; Falomir-Lockhart et al., 2019). Although SCFAs can be measured in circulation in perinatal mice (Kimura et al., 2020), and monocarboxylate transporters are expressed by capillary endothelial cells forming the BBB of newborn rodents (Omori et al., 2020), it has yet to be directly demonstrated whether SCFA signaling occurs in the newborn brain.

### Gut Hormones

Gut peptides, such as cholecystokinin, peptide YY, gastric inhibitory peptide, and ghrelin are produced by EECs and other gastrointestinal cells. The microbiota regulates the levels of these gut peptide hormones, which activate receptors on vagal afferents in the intestinal mucosa (Lach et al., 2018) or in the brain to affect neural circuits controlling eating behavior (Andermann and Lowell, 2017). Gut peptide hormones are measurable prenatally and approach adult levels by birth (Bryant et al., 1982); whether the release of these hormones is affected by the arrival of the pioneer microbiota in newborns is another area ripe for investigation.

### Immune Signaling

Immune signaling is a fourth route of microbiota-gut-brain signaling. For example, the innate immune receptors toll-like receptor (TLR) 4 and TLR5 are activated by the bacterial antigens lipopolysaccharide and flagellin, respectively. Both receptors are expressed at high levels and are fully functional in gut epithelial cells of perinatal mice (Gribar et al., 2009; Fulde et al., 2018). TLR5 selects against flagellated bacteria during the neonatal period (Fulde et al., 2018) and TLR4 binding in epithelial cells causes the release of proinflammatory cytokines (Gribar et al., 2009). In adults, this cytokine release activates brain regions such as the PVN (Rivest, 2001). Assuming that cytokines circulating in neonates also signal to the brain, this system may be in place at birth to detect the earliest-arriving microbes. In addition, TLRs are expressed in the perinatal mouse brain (e.g., Kaul et al., 2012), which may allow for direct brain sensing of bacteria-related molecules, although we currently know very little about their regional distribution in the perinatal brain.

## The Pioneer Microbiota – When Do They Arrive and “Who” Are They?

The newborn begins its lifelong exposure to gut microbes with its first swallow. Low levels of bacteria are detected in the gastric aspirate of human babies within 1 h of a vaginal but not a C-section birth, suggesting that these bacteria are acquired during passage through the birth canal (Bajorek et al., 2019). Almost immediately after birth the baby begins feeding, leading to the ingestion of almost one million bacteria daily from breast milk (Le Doare et al., 2018), although how bacteria get into the milk remains controversial (Greer et al., 2019). Microbes are present in human meconium samples on the first postnatal day (Hansen et al., 2015), and there is already evidence of a nascent microbiome in the lower intestinal tract of mice within a few hours of birth (van Best et al., 2020).

One might reasonably question whether the tiny numbers of microbes present in the first hours of life have any meaningful effect on the host, but two observations suggest that they may. In the Hawaiian bobtail squid, the development of the light-emitting organ is dependent on a specific bacterial species (*Vibrio fischeri*) entering the organ during a sensitive period. Remarkably, the bacteria populate the organ within minutes of hatching, and as few as 5 individual bacteria are sufficient to trigger its development (Altura et al., 2013). In mammals, the earliest functional effects of postnatal microbes reported to date may be the microbe-dependent activation of intestinal epithelial cells that is seen just 2 h after birth in mice (Lotz et al., 2006). This activation resolves several hours later, as the epithelial cells achieve tolerance. Interestingly, tolerance does not occur in mice born by sterile C-section and isolated from the dam (Lotz et al., 2006), demonstrating the requirement for direct exposure to microbes for this important process. This work also speaks to the “sterile womb” hypothesis: if bacteria are present in the fetal intestine, they are not numerous enough to trigger the activation of the intestinal epithelium that occurs just 2 h after exiting the womb.

Human and mouse studies have sought to characterize the pioneering gut bacteria in the first days of life. Pantoja-Feliciano et al. (2013) report that the earliest colonization of the mouse gut begins with the presence of *Streptococcus* 1 day after birth, followed by dominance of *Lactobacillus* (responsible for fermenting milk lactose) by day 3. The neonate transitions to a more stable gut microbial community dominated by *Bacteroides* around the time of weaning. This work largely replicates the findings made over 45 years ago by Schaedler (1973) who used culture techniques to characterize the pioneer microbiota in mice. Human studies have classified a similar transition in the first days of life from aerobic species (including *Streptococcus*) to a more diverse microbial profile including bacteria with varying oxygen requirements, including anaerobic or facultative anaerobic species (e.g., *Lactobacillus*), though this is then followed by dominance of *Bifidobacterium* by 1 week of life (Fanaro et al., 2003).

Thus, gut microbes are present within the first few hours of life, and a succession of dominating genera takes place in neonatal mice and humans. Moreover, these bacteria, even in very small numbers, can have an effect on the periphery. In the last 2–3 years, effects of these pioneer microbes on brain development have also been reported.

## How Does the Pioneer Microbiota Impact Brain Development?

The microbiota colonizes the newborn’s body during a time when the brain is being shaped by key developmental processes. In mice, these include colonization of the brain by microglia and developmental neuronal cell death. Microglia, the resident immune cells of the brain, increase markedly in number, and change in morphology and gene expression during the early postnatal period in mice (Nikodemova et al., 2015). Similarly, cell death, which eliminates roughly 50% of post-mitotic neurons via apoptosis, is concentrated during the first postnatal week in mice (Ahern et al., 2013). Neuronal cell death shows abrupt changes following birth (Mosley et al., 2017) which led us to hypothesize that microbiota colonization at birth may play a role in shaping this process.

Using GF mice, we found that microbiota absence at birth is associated with region-specific changes in cell death and increased microglial labeling in the hippocampus and hypothalamus (Castillo-Ruiz et al., 2018; Figure 1). These effects were not seen prenatally, but occurred within 12–14 h of birth, suggesting that direct exposure to microbes is necessary. We also found that the expression of pro-inflammatory cytokines, especially interleukin 1β and tumor necrosis factor α, was markedly higher in the brains of mice born in the presence of a microbiota than in those born GF (Castillo-Ruiz et al., 2018). More recently, we reported neural activation 3 h after birth in the PVN (Hoffiz et al., 2021), a brain region that receives input from the vagus nerve and has a central role in immune regulation and the stress response. This timing coincides with the arrival of microbiota to the newborn gut, although we have yet to demonstrate that the two events are causally linked. Since oral gavage with pathogenic bacteria causes activation of the PVN within 2 h in adults (Wang et al., 2002), it is certainly possible that pioneer bacteria could signal to the PVN very rapidly postpartum, and this is an important area for future study.

Other neurodevelopmental processes may be affected by microbiota colonization at birth in mice and in other species. The stage of brain development at birth varies between short- and long-gestation species, so the neural processes affected by the first exposure to a microbiota are also likely to vary. In humans, for example, cell death is ongoing at birth, but microglial colonization occurs prenatally (Menassa and Gomez-Nicola, 2018).

## Do Effects of the Microbiota Begin *In Utero*?

**FIGURE 2:**
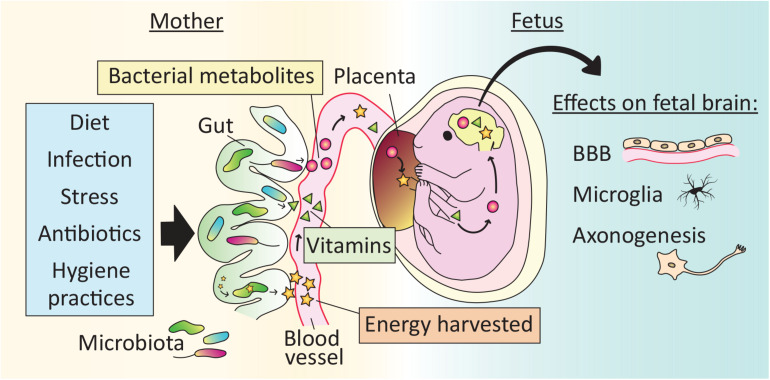
Effects of the microbiota on brain development begin *in utero*. The maternal gut microbiota exerts effects on fetal brain development indirectly via the production of vitamins and bacterial metabolites, as well as by increasing the energy harvested from food. Microbe-dependent molecules cross the placenta and may reach the fetal brain. Reported effects of the maternal microbiota on fetal brain development include development of the blood brain barrier (BBB), microglial cell number and physiology, and axonogenesis. As a result, perturbations to the maternal microbiota, for example via lifestyle (e.g., diet), illness (e.g., stress or infection) or medical treatment (e.g., antibiotics), may affect offspring brain development.

Regardless of the final outcome of the “sterile womb” debate, evidence is strong that microbes play an essential role in fetal development, including brain development, via *indirect* effects through the mother (Figure 2).

### Maternal Bacterial Metabolites Reach the Fetus

In mammals, all calories and nutrients required for fetal growth are transferred from the mother, and the microbiota influences this transfer in several ways. For example, the maternal gut microbiota increases the energy harvested from food, and is essential for the synthesis of vitamins and the generation of SCFAs (Macpherson et al., 2017). Recent evidence demonstrates that molecules derived from the maternal microbiota reach the fetus to influence gene expression and anatomical development of the brain.

In a global metabolomic analysis, Li et al. (2020) found that roughly 5% of the metabolites present in human fetal intestine and meconium could be classified as “microbial” (i.e., metabolites that are either produced by microbes or produced by the host in response to microbes). Because they did not detect any microbial signature in the fetus itself, the authors hypothesize that the microbial metabolites are vertically transmitted from the mother. Similarly, molecules from isotope-labeled bacteria administered to pregnant mouse dams reach the placenta and fetal circulation (Gomez de Agüero et al., 2016). In addition, when GF dams are transiently colonized with a genetically engineered strain of *E. coli* that does not persist in the intestine passed term, their offspring have more innate leukocytes and altered intestinal gene expression compared to pups born to unmanipulated GF dams (Gomez de Agüero et al., 2016). Some of the changes in the offspring of transiently colonized dams are related to the expression of antibacterial peptides and mucus production, and pups of gestation-only colonized mothers are better adapted to microbial challenges later in life. Thus, indirect effects of the maternal microbiota may prepare the fetus for the direct exposure to microbes encountered at birth.

### Maternal Microbiota-Dependent Metabolites Affect Brain and Peripheral Nervous System Development of Offspring

Fetuses gestating in GF dams have reduced expression of endothelial tight junction proteins and increased BBB permeability compared to fetuses of conventional control dams (Braniste et al., 2014), indicating that the maternal microbiota influences *in utero* development of the BBB. The microglia in male mouse embryos of GF mothers are more numerous and highly branched, and show substantial changes in gene expression compared to those of controls (Thion et al., 2018), demonstrating that development of the brain’s innate immune system is also affected by maternal microbial status.

Mouse embryos from antibiotic-treated and GF dams exhibit reduced thalamo-cortical axon growth (Vuong et al., 2020). This abnormality is due to a reduction in maternal microbiota-dependent metabolites reaching the fetus, and is prevented by colonizing the dams with even a limited set of bacteria (Vuong et al., 2020). Development of the peripheral nervous system is also affected by maternal microbial metabolites that reach the offspring *in utero*. For example, radioactively labeled SCFAs administered to the maternal colon reach their embryos within 40 min, and embryos lacking specific free fatty acid receptors have reduced development of sympathetic nerve projections to the heart (Kimura et al., 2020).

Thus, microbial molecules and microbe-dependent metabolites from the mother affect offspring neural development *in utero* (Figure 2). Koren et al. (2012) and others have reported dramatic changes in the maternal gut microbiota over the course of pregnancy, which suggests that the metabolites reaching the offspring may also vary during gestation. However, other studies report relatively stable microbial communities over pregnancy (e.g., Yang et al., 2020). The reason for the discrepancy is unclear, but could include differences in subject-related factors across studies, such as maternal age and body weight.

## Common Perturbations of the Microbiota That May Affect Offspring Brain Development

Although humans are never born GF (but see: Barnes et al., 1969), many variables of modern life can profoundly alter the maternal and newborn microbiota (Figure 2). Birth mode, birth timing, maternal infection, antibiotic exposure, breast milk vs. formula feeding, hygiene practices, maternal diet, and maternal stress all cause changes in the maternal and/or offspring microbiota and, in some cases, have been linked to behavioral or brain alterations in the offspring (Cryan et al., 2019; Jašarević and Bale, 2019).

For example, the offspring of female mice fed a high-fat diet before and during pregnancy have social deficits that are attributable to alterations in their microbiota (Buffington et al., 2016). Maternal infection during pregnancy influences offspring cortical development and later behavior in mice, and these effects appear to be mediated via the maternal microbiota (Kim et al., 2017). A staggering 40% of all United States women (and essentially 100% of those undergoing C-section) are now treated with antibiotics immediately prior to delivery (Kuperman and Koren, 2016), with demonstrated profound and surprisingly long-lasting effects on the gut microbiota of the newborn. In boys, neonatal antibiotic treatment impairs growth during at least the first 6 years of life, whereas treatment later in infancy does not (Uzan-Yulzari et al., 2021). Although this latter study was not designed to examine neurological outcomes, restricted childhood growth has previously been associated with poor neurodevelopment (Castanys-Muñoz et al., 2017). These findings, combined with the recent data showing effects of the very early microbiota on normal offspring brain development (section “How Does The Pioneer Microbiota Impact Brain Development?”), suggest that we should be cautious about clinical manipulations that alter the microbiota of newborns. In cases where such manipulations are unavoidable (e.g., antibiotic treatment of newborns with proven infections), knowledge of what microbes support normal neonatal brain development – and when – may allow us to intervene with therapeutic benefit.

## What’s Next?

It should not come as a surprise that microbes affect the developing brain, since at no time in evolutionary history has a nervous system developed in the absence of signals from microbes. Thus, the mammalian brain may have evolved to be “microbe expectant” in the same way that sensory systems are activity dependent, and require afferent input for normal development. If so, then both the presence and the relative absence of microbes can be potent signals, and interesting hypotheses are suggested for future study. We wonder, for example, whether the “leakiness” of the BBB seen in GF mice (e.g., Braniste et al., 2014) might be due to a feedback mechanism attempting to increase the availability of the (missing) signals from microbes.

Although the effects of the gut microbiota on brain development have been the subject of several published reviews, a careful reading of the literature reveals that few studies have actually examined the developing brain. Instead, effects of microbes on neurodevelopment are largely inferred from studies examining brain chemistry or behavior in adulthood. Observations in adults have been important in establishing long-term, functional consequences of the microbiota on the brain and behavior, but shed little light on exactly how the microbiota influences the developing brain. It is unknown, for example, which brain changes reflect primary responses to the microbiota and which are secondary effects of altered development elsewhere in the nervous system. Our ability to identify how the microbiota affects brain development will be hampered if we ignore the earliest, most direct effects, and the recent studies we have highlighted here are finally addressing this.

Many gaps in our understanding remain, however, including the pathways by which the very earliest microbes signal to the brain. Recent advances in metabolomics, in particular, promise to soon provide a more comprehensive picture of microbial metabolites that mediate effects of the maternal or newborn microbiota on brain development. Research in the gut-brain-axis field is progressing very rapidly, and is likely to soon illuminate this and other questions.

## Author Contributions

AG, NMR, AC-R, and NGF conceived, wrote, and edited the manuscript. BC provided useful insights and edited the manuscript. All authors contributed to the article and approved the submitted version.

## Conflict of Interest

The authors declare that the research was conducted in the absence of any commercial or financial relationships that could be construed as a potential conflict of interest.

## References

[B1] Ahern T. H. Krug S. Carr A. V. Murray E. K. Fitzpatrick E. Bengston L. (2013). Cell death atlas of the postnatal mouse ventral forebrain and hypothalamus: effects of age and sex. *J. Comp. Neurol.* 521 2551–2569. 10.1002/cne.23298 23296992 PMC4968939

[B2] Altura M. A. Heath-Heckman E. A. C. Gillette A. Kremer N. Krachler A.-M. Brennan C. (2013). The first engagement of partners in the *Euprymna scolopes*-*Vibrio fischeri* symbiosis is a two-step process initiated by a few environmental symbiont cells. *Environ. Microbiol.* 15 2937–2950. 10.1111/1462-2920.12179 23819708 PMC3937295

[B3] Andermann M. L. Lowell B. B. (2017). Towards a wiring-diagram understanding of appetite control. *Neuron* 95 757–778. 10.1016/j.neuron.2017.06.014 28817798 PMC5657399

[B4] Arpaia N. Campbell C. Fan X. Dikiy S. van der Veeken J. deRoos P. (2013). Metabolites produced by commensal bacteria promote peripheral regulatory T-cell generation. *Nature* 504 451–455. 10.1038/nature12726 24226773 PMC3869884

[B5] Bajorek S. Parker L. Li N. Winglee K. Weaver M. Johnson J. (2019). Initial microbial community of the neonatal stomach immediately after birth. *Gut Microbes* 10 289–297. 10.1080/19490976.2018.1520578 30404568 PMC6546338

[B6] Barnes R. D. Bentovim A. Hensman S. Piesowicz A. T. (1969). Care and observation of a germ-free neonate. *Arch. Dis. Child.* 44 211–217. 5813612 10.1136/adc.44.234.211 PMC2020043

[B7] Berni Canani R. Di Costanzo M. Leone L. (2012). The epigenetic effects of butyrate: potential therapeutic implications for clinical practice. *Clin. Epigenetics* 4:4. 10.1186/1868-7083-4-4 22414433 PMC3312834

[B8] Braniste V. Al-Asmakh M. Kowal C. Anuar F. Abbaspour A. Toth M. (2014). The gut microbiota influences blood-brain barrier permeability in mice. *Sci. Transl. Med.* 6:263ra158. 10.1126/scitranslmed.3009759 25411471 PMC4396848

[B9] Browning K. N. Verheijden S. Boeckxstaens G. E. (2017). The vagus nerve in appetite regulation, mood and intestinal inflammation. *Gastroenterology* 152 730–744. 10.1053/j.gastro.2016.10.046 27988382 PMC5337130

[B10] Bryant M. G. Buchan A. M. J. Gregor M. Ghatei M. A. Polak J. M. Bloom S. R. (1982). Development of intestinal regulatory peptides in the human fetus. *Gastroenterology* 83 47–54. 10.1016/S0016-5085(82)80283-7 7042452

[B11] Buchanan K. L. Bohórquez D. V. (2018). You are what you (first) eat. *Front. Hum. Neurosci.* 12:323. 10.3389/fnhum.2018.00323 30150928 PMC6099179

[B12] Buffington S. A. Di Prisco G. V. Auchtung T. A. Ajami N. J. Petrosino J. F. Costa-Mattioli M. (2016). Microbial reconstitution reverses maternal diet-induced social and synaptic deficits in offspring. *Cell* 165 1762–1775. 10.1016/j.cell.2016.06.001 27315483 PMC5102250

[B13] Castanys-Muñoz E. Kennedy K. Castañeda-Gutiérrez E. Forsyth S. Godfrey K. M. Koletzko B. (2017). Systematic review indicates postnatal growth in term infants born small-for-gestational-age being associated with later neurocognitive and metabolic outcomes. *Acta Paediatr.* 106 1230–1238. 10.1111/apa.13868 28382722 PMC5507303

[B14] Castillo-Ruiz A. Mosley M. George A. J. Mussaji L. F. Fullerton E. F. Ruszkowski E. M. (2018). The microbiota influences cell death and microglial colonization in the perinatal mouse brain. *Brain Behav. Immun.* 67 218–229. 10.1016/j.bbi.2017.08.027 28890156 PMC5696094

[B15] Clarke G. Grenham S. Scully P. Fitzgerald P. Moloney R. D. Shanahan F. (2013). The microbiome-gut-brain axis during early life regulates the hippocampal serotonergic system in a sex-dependent manner. *Mol. Psychiatr.* 18 666–673. 10.1038/mp.2012.77 22688187

[B16] Cryan J. F. Dinan T. G. (2012). Mind-altering microorganisms: the impact of the gut microbiota on brain and behaviour. *Nat. Rev. Neurosci.* 13 701–712. 10.1038/nrn3346 22968153

[B17] Cryan J. F. O’Riordan K. J. Cowan C. S. M. Sandhu K. V. Bastiaanssen T. F. S. Boehme M. (2019). The microbiota-gut-brain axis. *Physiol. Rev.* 99 1877–2013. 10.1152/physrev.00018.2018 31460832

[B18] de Goffau M. C. Lager S. Sovio U. Gaccioli F. Cook E. Peacock S. J. (2019). Human placenta has no microbiome but can contain potential pathogens. *Nature* 572 329–334. 10.1038/s41586-019-1451-5 31367035 PMC6697540

[B19] Falomir-Lockhart L. J. Cavazzutti G. F. Giménez E. Toscani A. M. (2019). Fatty acid signaling mechanisms in neural cells: fatty acid receptors. *Front. Cell. Neurosci.* 13:162. 10.3389/fncel.2019.00162 31105530 PMC6491900

[B20] Fanaro S. Chierici R. Guerrini P. Vigi V. (2003). Intestinal microflora in early infancy: composition and development. *Acta Paediatr. Suppl.* 91 48–55. 10.1111/j.1651-2227.2003.tb00646.x 14599042

[B21] Fulde M. Sommer F. Chassaing B. van Vorst K. Dupont A. Hensel M. (2018). Neonatal selection by toll-like receptor 5 influences long-term gut microbiota composition. *Nature* 560 489–493. 10.1038/s41586-018-0395-5 30089902

[B22] Fülling C. Dinan T. G. Cryan J. F. (2019). Gut microbe to brain signaling: what happens in vagus. *Neuron* 101 998–1002. 10.1016/j.neuron.2019.02.008 30897366

[B23] Gomez de Agüero M. Ganal-Vonarburg S. C. Fuhrer T. Rupp S. Uchimura Y. Li H. (2016). The maternal microbiota drives early postnatal innate immune development. *Science* 351 1296–1302. 10.1126/science.aad2571 26989247

[B24] Greer F. R. Sicherer S. H. Burks A. W. Committee on Nutrition, and Section on Allergy and Immunology. (2019). The effects of early nutritional interventions on the development of atopic disease in infants and children: the role of maternal dietary restriction, breastfeeding, hydrolyzed formulas, and timing of introduction of allergenic complementary foods. *Pediatrics* 143:e20190281. 10.1542/peds.2019-0281 30886111

[B25] Gribar S. C. Sodhi C. P. Richardson W. M. Anand R. J. Gittes G. K. Branca M. F. (2009). Reciprocal expression and signaling of TLR4 and TLR9 in the pathogenesis and treatment of necrotizing enterocolitis. *J. Immunol.* 182 636–646. 10.4049/jimmunol.182.1.636 19109197 PMC3761063

[B26] Hansen R. Scott K. P. Khan S. Martin J. C. Berry S. H. Stevenson M. (2015). First-pass meconium samples from healthy term vaginally-delivered neonates: an analysis of the microbiota. *PLoS One* 10:e0133320. 10.1371/journal.pone.0133320 26218283 PMC4517813

[B27] Hoffiz Y. C. Castillo-Ruiz A. Hall M. A. L. Hite T. A. Gray J. M. Cisternas C. D. (2021). Birth elicits a conserved neuroendocrine response with implications for perinatal osmoregulation and neuronal cell death. *Sci. Rep.* 11:2335. 10.1038/s41598-021-81511-1 33504846 PMC7840942

[B28] Jašarević E. Bale T. L. (2019). Prenatal and postnatal contributions of the maternal microbiome on offspring programming. *Front. Neuroendocrinol.* 55:100797. 10.1016/j.yfrne.2019.100797 31574280

[B29] Kaelberer M. M. Buchanan K. L. Klein M. E. Barth B. B. Montoya M. M. Shen X. (2018). A gut-brain neural circuit for nutrient sensory transduction. *Science* 361:eaat5236. 10.1126/science.aat5236 30237325 PMC6417812

[B30] Kaul D. Habbel P. Derkow K. Krüger C. Franzoni E. Wulczyn F. G. (2012). Expression of toll-like receptors in the developing brain. *PLoS One* 7:e37767. 10.1371/journal.pone.0037767 22666391 PMC3364272

[B31] Kim S. Kim H. Yim Y. S. Ha S. Atarashi K. Tan T. G. (2017). Maternal gut bacteria promote neurodevelopmental abnormalities in mouse offspring. *Nature* 549 528–532. 10.1038/nature23910 28902840 PMC5870873

[B32] Kimura I. Miyamoto J. Ohue-Kitano R. Watanabe K. Yamada T. Onuki M. (2020). Maternal gut microbiota in pregnancy influences offspring metabolic phenotype in mice. *Science* 367:eaaw8429. 10.1126/science.aaw8429 32108090

[B33] Koren O. Goodrich J. K. Cullender T. C. Spor A. Laitinen K. Bäckhed H. K. (2012). Host remodeling of the gut microbiome and metabolic changes during pregnancy. *Cell* 150 470–480. 10.1016/j.cell.2012.07.008 22863002 PMC3505857

[B34] Kuperman A. A. Koren O. (2016). Antibiotic use during pregnancy: how bad is it? *BMC Med.* 14:91. 10.1186/s12916-016-0636-0 27312712 PMC4911692

[B35] Lach G. Schellekens H. Dinan T. G. Cryan J. F. (2018). Anxiety, depression, and the microbiome: a role for gut peptides. *Neurotherapeutics* 15 36–59. 10.1007/s13311-017-0585-0 29134359 PMC5794698

[B36] Le Doare K. Holder B. Bassett A. Pannaraj P. S. (2018). Mother’s milk: a purposeful contribution to the development of the infant microbiota and immunity. *Front. Immunol.* 9:361. 10.3389/fimmu.2018.00361 29599768 PMC5863526

[B37] Li Y. Toothaker J. M. Ben-Simon S. Ozeri L. Schweitzer R. McCourt B. T. (2020). In utero human intestine harbors unique metabolome, including bacterial metabolites. *JCI Insights* 5:e138751. 10.1172/jci.insight.138751 33001863 PMC7710283

[B38] Lotz M. Gütle D. Walther S. Ménard S. Bogdan C. Hornef M. W. (2006). Postnatal acquisition of endotoxin tolerance in intestinal epithelial cells. *J. Exp. Med.* 203 973–984. 10.1084/jem.20050625 16606665 PMC2118301

[B39] Macpherson A. J. de Agüero M. G. Ganal-Vonarburg S. C. (2017). How nutrition and the maternal microbiota shape the neonatal immune system. *Nat. Rev. Immunol.* 17 508–517. 10.1038/nri.2017.58 28604736

[B40] Menassa D. A. Gomez-Nicola D. (2018). Microglial dynamics during human brain development. *Front. Immunol.* 9:1014. 10.3389/fimmu.2018.01014 29881376 PMC5976733

[B41] Mosley M. Shah C. Morse K. A. Miloro S. A. Holmes M. M. Ahern T. H. (2017). Patterns of cell death in the perinatal mouse forebrain. *J. Comp. Neurol.* 525 47–64. 10.1002/cne.24041 27199256 PMC5116296

[B42] Nikodemova M. Kimyon R. S. De I. Small A. L. Collier L. S. Watters J. J. (2015). Microglial numbers attain adult levels after undergoing a rapid decrease in cell number in the third postnatal week. *J. Neuroimmunol.* 278 280–288. 10.1016/j.jneuroim.2014.11.018 25468773 PMC4297717

[B43] Omori K. Tachikawa M. Hirose S. Taii A. Akanuma S. Hosoya K. (2020). Developmental changes in transporter and receptor protein expression levels at the rat blood-brain barrier based on quantitative targeted absolute proteomics. *Drug Metab. Pharmacok.* 35 117–123. 10.1016/j.dmpk.2019.09.003 31974045

[B44] Pantoja-Feliciano I. G. Clemente J. C. Costello E. K. Perez M. E. Blaser M. J. Knight R. (2013). Biphasic assembly of the murine intestinal microbiota during early development. *ISME J.* 7 1112–1115. 10.1038/ismej.2013.15 23535917 PMC3660675

[B45] Penkova N. I. Baltadjiev G. A. Koeva Y. A. Atanassova P. K. Andonov V. N. Trichkova V. A. (2010). Prenatal and postnatal differentiation of small intestine in rat. *Folia Med.* 52 54–62. 20380288

[B46] Rackaityte E. Halkias J. Fukui E. M. Mendoza V. F. Hayzelden C. Crawford E. D. (2020). Viable bacterial colonization is highly limited in the human intestine in utero. *Nat. Med.* 26 599–607. 10.1038/s41591-020-0761-3 32094926 PMC8110246

[B47] Ratcliffe E. M. Fan L. Mohammed T. J. Anderson M. Chalazonitis A. Gershon M. D. (2011). Enteric neurons synthesize netrins and are essential for the development of the vagal sensory innervation of the fetal gut. *Dev. Neurobiol.* 71 362–373. 10.1002/dneu.20869 21485011 PMC3160128

[B48] Rivest S. (2001). How circulating cytokines trigger the neural circuits that control the hypothalamic-pituitary-adrenal axis. *Psychoneuroendocrinology* 26 761–788. 10.1016/s0306-4530(01)00064-6 11585678

[B49] Schaedler R. W. (1973). The relationship between the host and its intestinal microflora. *Proc. Nutr. Soc.* 32 41–47. 10.1079/PNS19730013 4598409

[B50] Silva Y. P. Bernardi A. Frozza R. L. (2020). The role of short-chain fatty acids from gut microbiota in gut-brain communication. *Front. Endocrinol.* 11:25. 10.3389/fendo.2020.00025 32082260 PMC7005631

[B51] Thion M. S. Low D. Silvin A. Chen J. Grisel P. Schulte-Schrepping J. (2018). Microbiome influences prenatal and adult microglia in a sex-specific manner. *Cell* 172 500–516.e16. 10.1016/j.cell.2017.11.042 29275859 PMC5786503

[B52] Uchimura Y. Fuhrer T. Li H. Lawson M. A. Zimmermann M. Yilmaz B. (2018). Antibodies set boundaries limiting microbial metabolite penetration and the resultant mammalian host response. *Immunity* 49 545–559.e5. 10.1016/j.immuni.2018.08.004 30193848 PMC6162337

[B53] Uzan-Yulzari A. Turta O. Belogolovski A. Ziv O. Kunz C. Perschbacher S. (2021). Neonatal antibiotic exposure impairs child growth during the first six years of life by perturbing intestinal microbial colonization. *Nat. Commun.* 12:443. 10.1038/s41467-020-20495-4 33500411 PMC7838415

[B54] van Best N. Rolle-Kampczyk U. Schaap F. G. Basic M. Olde Damink S. W. M. Bleich A. (2020). Bile acids drive the newborn’s gut microbiota maturation. *Nat. Commun.* 11:3692. 10.1038/s41467-020-17183-8 32703946 PMC7378201

[B55] Vuong H. E. Pronovost G. N. Williams D. W. Coley E. J. L. Siegler E. L. Qiu A. (2020). The maternal microbiome modulates fetal neurodevelopment in mice. *Nature* 586 281–286. 10.1038/s41586-020-2745-3 32968276 PMC7554197

[B56] Walter J. Hornef M. W. (2021). A philosophical perspective on the prenatal in utero microbiome debate. *Microbiome* 9:5. 10.1186/s40168-020-00979-7 33436093 PMC7805158

[B57] Wang D. Coscoy L. Zylberberg M. Avila P. C. Boushey H. A. Ganem D. (2002). Microarray-based detection and genotyping of viral pathogens. *Proc. Natl. Acad. Sci. U.S.A.* 99 15687–15692. 10.1073/pnas.242579699 12429852 PMC137777

[B58] Yang H. Guo R. Li S. Liang F. Tian C. Zhao X. (2020). Systematic analysis of gut microbiota in pregnant women and its correlations with individual heterogeneity. *NPJ Biofilms Microbiomes* 6 1–12. 10.1038/s41522-020-00142-y 32917878 PMC7486914

